# Multicenter investigation of pediatric gastrointestinal tract magnets ingestion in China

**DOI:** 10.1186/s12887-020-1990-9

**Published:** 2020-02-28

**Authors:** Kai Wang, Dan Zhang, Xianling Li, Zengmeng Wang, Guangjun Hou, Xinjian Jia, Huizhong Niu, Shiqin Qi, Qingqiang Deng, Bin Jiang, Hongqiang Bian, Heying Yang, Yajun Chen

**Affiliations:** 1 Department of General Surgery, Beijing Children’s Hospital, Capital Medical University, National Center for Children’s Health, No.56 Nanlishi St, Xicheng District, Beijing, 100045 China; 2 Department of General Surgery, Zhengzhou Children’s Hospital, Zhengzhou, 450053 Henan China; 3 Department of General Surgery, Xi’an Children’s Hospital, Xi’an, 710043 Shaanxi China; 4 Department of General Surgery, Children’s Hospital of Hebei Province, Shijiazhuang, 050030 Hebei China; 5 Department of General Surgery, Anhui Provincial Children’s Hospital, Hefei, 340111 Anhui China; 6 Department of General Surgery, Jiangxi Provincial Children’s Hospital, Nanchang, 330006 Jiangxi China; 7 Department of General Surgery, Children’s Hospital of Nanjing Medical University, Nanjing, 210008 China; 8 Department of General Surgery, Wuhan Children’s Hospital, Wuhan, 430015 Hubei China; 9 Department of Pediatric Surgery, the First Affiliated Hospital of Zhengzhou University, Zhengzhou, 450052 Henan China

**Keywords:** Pediatric, Gastrointestinal tract, Magnetic foreign body, Buckyball

## Abstract

**Purpose:**

To describe the incidence and management of gastrointestinal tract Buckyball magnets ingestions in a multicenter Chinese pediatric patient population, and discuss the preventive measures.

**Methods:**

Medical records of 74 pediatric patients from 9 large Chinese hospitals during the past 10 years, who were diagnosed as buckyball magnets ingestion and got invasive treatment, were retrospectively studied. The follow-up was through telephone and outpatient service to estimate the post-surgery condition. Information collection was through online questionnaire.

**Results:**

Among the 74 cases, there were 50 boys (68%) and 24 girls (32%). The median age was 36 (interquartile range (IQR) 22–77) months, with a range of 7 months to 11 years, and it showed two peaks, the first between 1 and 3 years, and the second between 6 to 11 years. The annual case number showed a sharp increase over time, and the total case number in the last 2 years (2017 and 2018) showed a greater than 9-fold increase when compared with the first 2 years (2013 and 2014). The majority of ingestions were unintentional, with only 3 patients deliberately swallowing the Buckyball magnets. The median time of ingestion until the onset of emergent symptoms was 2 (IQR 1–5) days, and ranged from 4 h to 40 days. Twenty-one patients had no symptoms, and the remaining cases presented with abdominal pain, vomiting, fever, abdominal distension, excessive crying, melena, and the ceasing of flatus and defecation. Gastroscopy, colonoscopy, laparoscopic surgery and laparotomy surgery were performed in accordance with the algorithm from the North American Society of Pediatric Gastroenterology, Hepatology and Nutrition (NASPGHAN). Procedural and operative findings included gastrointestinal mucosa erosion, ischemia and necrosis, perforation, and abdominal abscess, fistula and intestinal obstruction. The median number of Buckyball magnets ingested was 4 (IQR 2–8), with a range from 1 to 39. During the median follow-up period of 6 (IQR 1–15) months, 3 patients had intestinal obstruction, and one underwent a second operation. The remaining 71 patients courses were uneventful during the follow-up period. None of the 74 patients reported a second swallowing of foreign bodies.

**Conclusions:**

The incidence of pediatric gastrointestinal tract magnets ingestion in China is increasing. Management of such patients should follow the NASPGHAN algorithm. Preventive measures to limit children’s access to Buckyball magnets should be taken from three levels, namely the national administration, producer, and consumer.

## Background

Alimentary tract foreign body ingestion is common in children [1]. Research showed that 80% of the ingestions cause no harm, with the foreign body being passed out of the alimentary tract without incident [2]. However, magnetic foreign bodies are a special type of foreign body, as they can cause severe injuries to the gastrointestinal tract, and even be life-threatening. When swallowed alone, they tend to pass through the gastrointestinal tract uneventfully. However, if multiple magnetic foreign bodies are swallowed, with or without metal foreign body, they can attract each other across layers of intestines, and cause ischemia, pressure necrosis, perforation and volvulus of the intestines, leading to severe illness.

Buckyball, also known as magical magnet, is a special kind of toy, which is made of rare-earth magnets. Unlike traditional magnets, Buckyball is small in volume but powerful in magnetism, and easily swallowed by children. The severity of pediatric injuries from magnets ingestion in the United States had been investigated by NASPGHAN, and the algorithm guided for the diagnosis and treatment for magnets ingestion, including Buckyball magnets, was published in 2012 [3, 4]. Faced with the increase of Buckyball-related gastrointestinal injuries in children, after years of efforts, the United States government finally held a recall for Buckyballs in 2014, and the incidence has sharply declined since then [5]. However, unlike the United States, it has recently become more popular in China, and pediatric gastrointestinal injuries due to Buckyballs appear to show a corresponding increase. To date, no large-scale case study has analyzed or summarized the incidence and severity of gastrointestinal Buckyball injuries in Chinese pediatric patients.

Thus, the purpose of the study is to describe the incidence and management of gastrointestinal tract Buckyball ingestion in Chinese pediatric patients, and discuss the preventive measures toward the issue through a multicenter investigation.

## Methods

### Patients

All the pediatric patients from January 2009 to March 2019, who were under the age of 18 years old, diagnosed with gastrointestinal tract Buckyball ingestion, admitted to emergency department, and underwent endoscopy or surgical intervention, were included in this study. The patients were from 9 large Chinese hospitals, among which 8 were pediatric hospitals, including Beijing Children’s Hospital, Zhengzhou Children’s Hospital, Xi’an Children’s Hospital, Children’s Hospital of Hebei Province, Anhui Provincial Children’s Hospital, Jiangxi Provincial Children’s Hospital, Children’s Hospital of Nanjing Medical University, and Wuhan Children’s Hospital. The remaining hospital was a general hospital, the First Affiliated Hospital of Zhengzhou University.

### Data collection

Questionnaires were distributed online, medical records of the patients were reviewed retrospectively and special attention was given regarding the age, gender, clinical manifestations, treatments of the patients, and number and site of Buckyball. Post-surgery follow-up was through telephone and outpatient service. The follow-up was from the initial injury to the end of the study, which is June 2019.

### Statistical analysis

All the data was analyzed using SPSS for Windows version 17.0. Normal distribution data was presented by (mean ± standard deviation), non-normal distribution data was presented by median (interquartile range (IQR) first quartile- third quartile), and categorical variables were presented by frequencies and percentages.

## Results

**Fig. 1:**
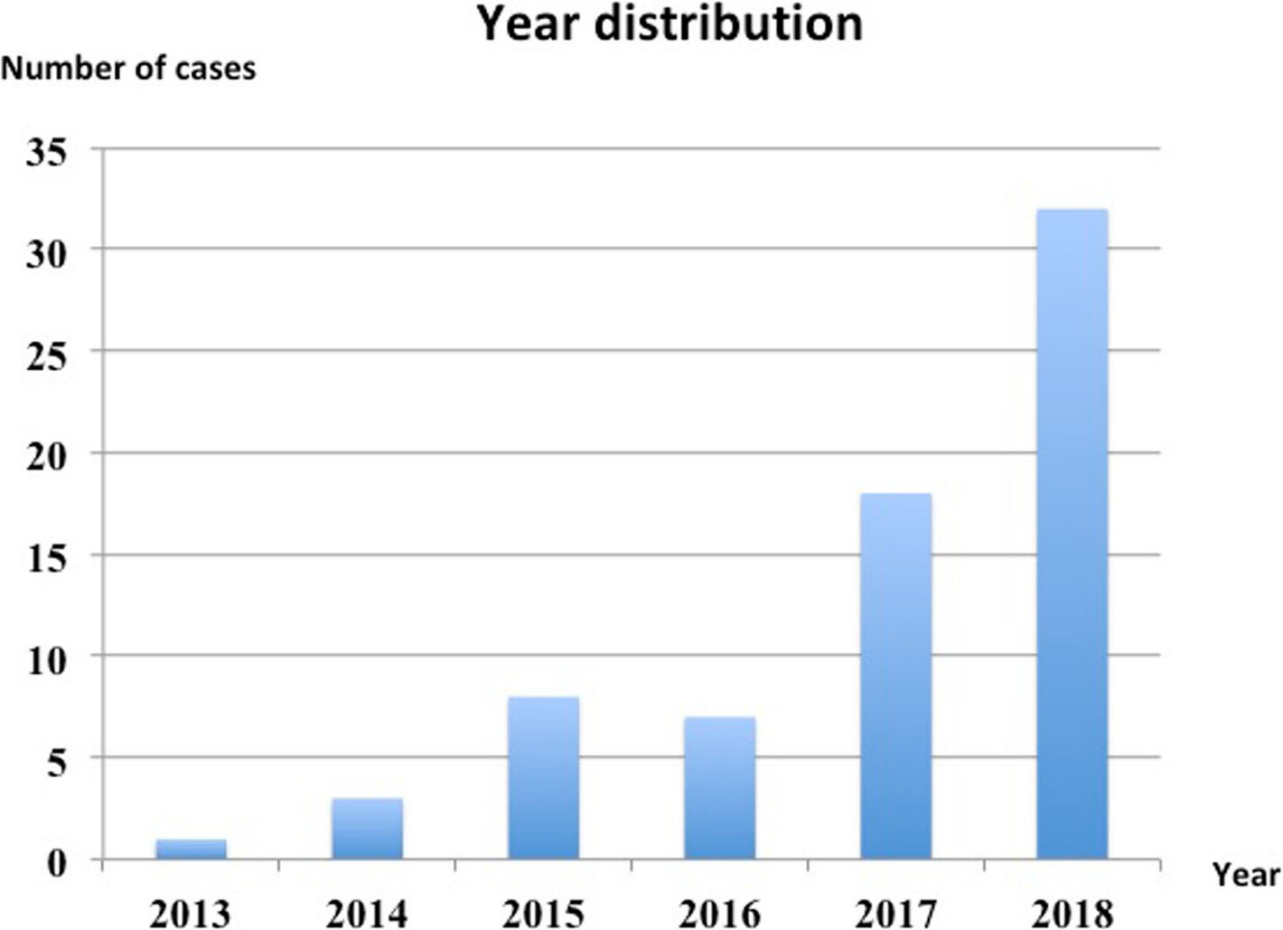
Results showed the year distribution of gastrointestinal Buckyball injuries

Seventy-four pediatric patients with gastrointestinal tract Buckyball injury were included in the study. Among the cases were 50 boys (68%) and 24 girls (32%), with an obvious gender prevalence towards boys (Table 1). The median age was 36 (IQR 22–77) months, ranging from 7 months to 11 years, and it showed two peaks, the first between 1 and 3 years, and the second between 6 to 11 years (Table 1). During the investigation period, the first case occurred in 2013. There was a sharp increase in the annual case number (Fig. 1) and the total case number in the last 2 years of our study (2017 and 2018) showed a greater than 9-fold increase when compared with the first 2 years (2013 and 2014). All 74 patients had no diagnosed psychological disorder or pica. Only 3 of them had specific reasons for swallowing the foreign body; a male infant was fed the buckyballs by his older cousin for his own amusement, a school-aged boy ingested the balls deliberately to demonstrate his bravery, another girl swallowed the Buckyballs because she mistook them for candy. The remaining 71 patients were playing with the Buckyballs and accidentally swallowed them.

**Table 1:** Demographics of the patients and the hospitals

Categories	Variables	Number	Frequency (%)
Gender	Male	50	68.0
	Female	24	32.0
Age group	0 ~ 0.5	0	0
	0.5 ~ 1	2	2.7
	1 ~ 3	35	47.3
	3 ~ 6	16	21.6
	6 ~ 11	19	25.7
	11 ~ 18	2	2.7
Hospital name	BCH	41	55.4
	ZCH	9	12.1
	XCH	6	8.1
	CHHP	5	6.7
	APCH	3	4.1
	JPCH	3	4.1
	CHNMU	3	4.1
	WCH	2	2.7
	FAHZU	2	2.7

*BCH* Beijing Children’s Hospital, *ZCH* Zhengzhou Children’s Hospital, *XCH* Xi’an Children’s Hospital, *CHHP* Children’s Hospital of Hebei Province, *APCH* Anhui Provincial Children’s Hospital, *JPCH* Jiangxi Provincial Children’s Hospital, *CHNMU* Children’s Hospital of Nanjing Medical University, *WCH* Wuhan Children’s Hospital, *FAHZU* the First Affiliated Hospital of Zhengzhou University

**Fig. 2:**
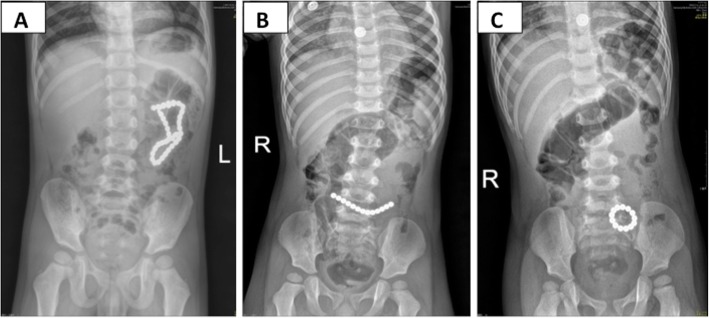
Select radiology images of ingested Buckyballs. The maximum of 39 Buckyballs ingested (**a**). Another patient ingested 13 Buckyballs, which showed line type on the first day (**b**), and turned to annular type on the following day (**c**)

**Fig. 3:**
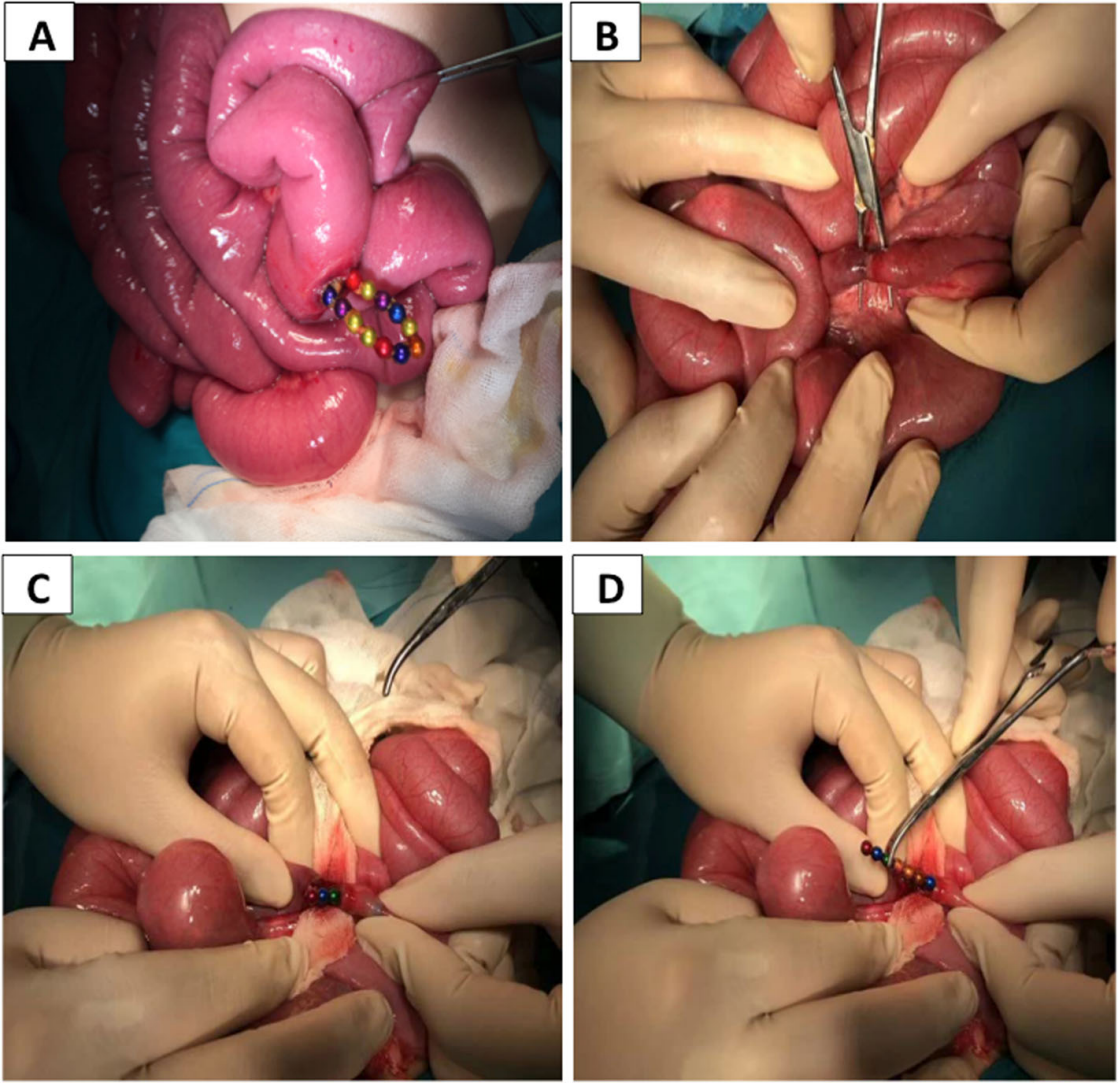
Intra-operative images for one patient. Buckyballs caused intestinal perforation (**a**) and fistula (**b**-**d**)

The median time of ingestion until the onset of emergent symptoms was 2 (IQR 1–5) days, and ranged from 4 h to 40 days. Twenty-one patients had no symptoms, but their parents or guardians had been alerted to the fact that they had swallowed the Buckyballs and sought medical help at the hospitals; other patients were symptomatic (Table 2). Radiological examination was useful to demonstrate Buckyball’s number and location (Fig. 2 A-C), which helped to guide further treatment. The patients underwent gastroscopy, colonoscopy, laparoscopic surgery and laparotomy surgery depending on the NASPGHAN algorithm (Table 3), that if the patient was asymptomatic, then endoscopy should be considered firstly, and if it failed, then operation should be performed. However, if the patient was symptomatic, or the magnets were multiple and beyond the stomach, then operation should be performed directly. Besides, if the patient had only one magnet, then wait-and-see strategy could be taken. During the surgery, gastrointestinal perforation (Fig. 3a), ischemia and necrosis of gastrointestinal wall, abscess, intestinal obstruction, fistula (Fig. 3b-d) and gastrointestinal mucosa erosion were found (Table 3). The median number of Buckyballs was 4 (IQR 2–8), with a range from 1 to 39.

**Table 2:** Symptoms of the patients

Symptoms	Number	Frequency (%)
No	21	28.4
Abdominal pain	40	54.1
Vomiting	36	48.7
Fever	11	14.9
Abdominal distension	3	4.1
Excessive crying	3	4.1
Melena	2	2.1
Flatus and defecation stopped	1	1.4

**Table 3:** Treatment procedures and findings during operation

Categories	Variables	Total number	Frequency (%)	Number of success
Treatment	Gastroscopy	16	21.6	7
	Colonoscopy	1	1.4	0
	Laparoscopic surgery	6	8.1	5
	Laparotomy surgery	41	55.4	41
	Conversion to laparotomy	10	13.5	10
Findings	Gastrointestinal perforation	38	51.4	–
	Ischemia and necrosis of gastrointestinal wall	14	19.0	–
	Abscess	7	9.5	–
	Intestinal obstruction	6	8.1	–
	Fistula	2	2.7	–
	Gastrointestinal mucosa erosion	28	37.8	–

During the follow-up period of 6 (IQR 1–15) months, three patients had intestinal obstruction after laparotomy surgery, and one of them underwent a second operation to relieve the obstruction. The remaining 71 patients had no abdominal distension, adhesive intestinal obstruction, or delayed perforation incident. None of the 74 patients reported a second swallowing of Buckyballs or other magnetic foreign bodies in the follow-up period.

## Discussion

Alimentary tract foreign body ingestion is common in pediatrics, especially in infants and toddlers [6]. Among them, more than 80% need no intervention and the foreign body will pass out uneventfully. Only 20% calls for further attention, and less than 1% requires surgery [2, 7]. Treatments are different for different categories of foreign bodies. The wait-and-see strategy can be used in the non-magnetic foreign body, such as coins, jewelries, and small plastic toys, when they are in the stomach and beyond. However for the remaining 20% patients who have fish bones, jujube pits, batteries, or magnets ingestions, active intervention is needed. Unlike other foreign bodies, ingested magnets can catch loops of intestine, which leads to gastrointestinal wall ischemia, pressure necrosis or perforation, and potential death [8, 9]. Other studies also described intestinal obstruction from internal hernia and volvulus of intestine, fistula formation [10], and hemorrhage when the mesenteric was involved [10]. Midget J reported a twenty-month old boy who had ingested magnets, causing intestinal necrosis and abdominal sepsis, which led to death [11, 12]. Additionally, Waters AM [13] reported another case of death due to the hemorrhage from an esophago-aortic fistula induced by ingestion of magnets. Thus, the risk of magnets ingestion remains higher than the ingestion of non-magnetic foreign bodies. An earlier study revealed that from 2003 to 2009, 38 cases of magnetic foreign body ingestion were identified, of which 8 cases were multiple magnet ingestion, and the number continues to grow [14]. Another investigation in 2013 reported that during 2002 and 2011, there had been more than 22,000 pediatric magnetic foreign body ingestion cases in America, and the investigation showed a 5-fold increase comparing the first and the last 2 years [4].

Rare-earth magnet, made from NdFeB (neodymium iron boron), a newly developed magnetic material, has a maximum magnetic energy force 5 to 10 times than that of the ordinary ferrite [12, 15, 16], suggesting that the tiny rare-earth magnet can produce a huge magnetic force. It was primarily developed for industrial manufacturation such as electrical machinery, medical apparatus and instruments, in order to decrease the volume of production and enlarge its properties. However, in recent years, it has been largely used in toy production. Buckyball, made of this type of magnet and mostly consisting of 216 magnets all 5 mm in size, is colorful and affordable. It is also able to activate thinking skills and motivate creativity. Since it was first introduced to the market in 2009 by Maxfield & Oberton [15, 17], it has been given to children of all ages, and is extremely addicting. Buckyball was initially labeled for ages over 13, but this was later changed to “keep away from all children” and the recommended age was adjusted to ages over 14, due to the 2010 definition change of “children” to “anyone under 14”. With its popularity, the number of horrifying gastrointestinal injuries in children increased [18]. Unlike ordinary magnets, Buckyballs can easily attract each other even through 6 layers of intestinal walls [19], with an average distance of 3.5 cm [20], and this strength increases when multiple Buckyballs are ingested [20]. In 2012, the consumer product safety commission (CPSC) in the United States sued Maxfield & Oberton demanding that Buckyballs be removed from store shelves, and that all existing products be removed. Although the company launched a resistance campaign, many retailers removed Buckyballs from their shelves and followed the CPSC action to recall the balls in 2014. Since the recall, a significant decrease was reported in multiple mini-magnet ingestion [5]. However, unlike the United States, our results (Fig. 1a) revealed that gastrointestinal tract Buckyball injuries in China continue to increase sharply annually, with no trend of decline.

Our investigation showed the peak age of the children who ingested the magnets was between 1 and 3 years old, and between 6 to 11 years old (Table 1), which was consistent with De Roo AC’s study in 2013 [21]. This may be explained by the fact that toddlers explore the world with their mouths and may accidentally swallow the Buckyballs when playing, and as a means for school-aged children to boast of their bravery, as reported by one patient in this study. However, 71 of the 74 patients ingested the Buckyballs unintentionally, a finding consistent with the reports from De Roo AC [21] and the NASPGHAN [6]. Other special reasons such as psychological disorders or pica [14, 15] were not found in this study. The ingestion displayed an obvious male predominance at any age period (Table 1), which was in accordance with other reports [22]. This may be attributed to the mischievous and curious nature of boys.

Beyond the rising popularity of Buckyball, severe gastrointestinal injuries occurred. Symptoms were not specific, and were dependent on the time of presentation and location of the Buckyballs, but more than half of the patients (40/74) presented with abdominal pain. Richard Sola Jr. concluded that abdominal pain was one risk factor for emergency surgery [23]. Other complications, such as ischemia and necrosis, gastrointestinal perforation (Fig. 3a), abscess, and gastrointestinal fistula (Fig. 3b-d) also proved to be critical. Diagnosis and treatment algorithm towards magnets ingestion were published by the NASPGHAN in 2012 [3], and such patients should be managed according to this guideline.

Although treatment is of great significance, prevention is much more important. China should take measures for prevention of Buckyball ingestion, and these suggestions listed should be followed. Firstly, on the national administration level, the production and trade of Buckyballs as well as other high-powered magnetic toys should be stopped [16]. The serious complications that could arise should also be publicized and reinforced by various media means. Secondly, on the production level, a national policy should be implemented, targeted towards pediatric toy productions. The magnetic toy size should be enlarged and the material used to make such toys should either revert back to the ordinary magnet that has low magnetic energy product [24] or use a magnetic force lowered to the flux index of 50 kG2 [12]. Warning labels should be much more prominent. Thirdly, on the consumer level, parents and caregivers should be made aware about the potential risk of the toy [11, 17, 24]; and children younger than 14 years old or who have psychological disorders and pica should refrain from playing with it. Parents should monitor their children more closely when playing with magnetic toys and also educate them about the right way to play with such toys [4, 7]. Additionally, children should be taught not only about the side effects of ingesting foreign objects, but also to stop instigating harmful ingestion among their peers. When evaluating the complaints of unexplained abdominal pain, clinicians should inquire specifically about the possible ingestions that a child might have made [16].

## Conclusion

The incidence of pediatric gastrointestinal tract magnetic foreign body ingestion in China is increasing. Management of such patients should follow the NASPGHAN algorithm. Preventive measures to limit children’s access to Buckyball magnets should be taken from three levels, namely the national administration, producer, and consumer.

## Data Availability

The data is available from the corresponding author on reasonable request.

## Abbreviations

CPSC: Consumer product safety commission
IQR: Interquartile range
NASPGHAN: North American Society of Pediatric Gastroenterology, Hepatology and Nutrition
NdFeB: Neodymium iron boron
